# On the number of genomic pacemakers: a geometric approach

**DOI:** 10.1186/s13015-014-0026-0

**Published:** 2014-12-31

**Authors:** Sagi Snir

**Affiliations:** Department of Evolutionary Biology, University of Haifa, Haifa, 31905 Israel

**Keywords:** Molecular evolution, Genome evolution pacemaker, Deming regression, Partition distance, Gap statistics

## Abstract

The universal pacemaker (UPM) model extends the classical molecular clock (MC) model, by allowing each gene, in addition to its individual intrinsic rate as in the MC, to accelerate or decelerate according to the universal pacemaker. Under UPM, the relative evolutionary rates of all genes remain nearly constant whereas the absolute rates can change arbitrarily. It was shown on several taxa groups spanning the entire tree of life that the UPM model describes the evolutionary process better than the MC model. In this work we provide a natural generalization to the UPM model that we denote multiple pacemakers (MPM). Under the MPM model every gene is still affected by a single pacemaker, however the number of pacemakers is not confined to one. Such a model induces a partition over the gene set where all the genes in one part are affected by the same pacemaker and task is to identify the pacemaker partition, or in other words, finding for each gene its associated pacemaker. We devise a novel heuristic procedure, relying on statistical and geometrical tools, to solve the problem and demonstrate by simulation that this approach can cope satisfactorily with considerable noise and realistic problem sizes. We applied this procedure to a set of over 2000 genes in 100 prokaryotes and demonstrated the significant existence of two pacemakers.

## Background

The Universal PaceMaker (UPM) of genome evolution [1] extends the classical Molecular Clock (MC) model [2] and its various imperative relaxations (see e.g. [3,4] among a few), by relaxing the rate constancy (as in MC) on one hand, and yet preserving the rate correlation between the various genes. Such a model can provide explanation to the striking phenomenon that the distribution of the evolutionary distances between orthologous genes remains remarkably constant across the entire history of life [5-7]. Under the UPM, all genes in each evolutionary lineage adhere to the pace of a pacemaker (PM), and change their evolutionary rate (approximately) in unison although the pacemaker’s pace at different lineages may differ. The UPM model is compatible with the large amount of data on fast-evolving and slow-evolving organismal lineages, primarily different groups of mammals [8]. Alternatively, the constancy of gene-specific relative rates is also an outcome of the MC model, under which the different genes evolve at roughly constant albeit different (gene-specific) rates. In a line of works [1,9,10] we established the superiority of the UPM model over the MC by explaining a larger fraction of the variance in the branch lengths of thousands of gene trees spanning the entire tree of life. Despite its relative simplicity, it was noted [11] that the UPM is “the most plausible model of genomic evolution and appears to have some statistical support”.

Although highly statistically significant, in absolute terms however, the advantage of UPM over MC was small, and both models exhibited considerable evolution rate overdispersion. A plausible explanation to the latter is that instead of a single, apparently weak (overdispersed) PM, there are independent *multiple pacemakers* that each affect a (different) subset of genes and are less dispersed than the single pacemaker. Throughout, we use the notation UPM to refer to the model and the PM term for the pacemaker as an object.

Primarily, we investigate the requirements for the identification of distinct PMs and assignment of each gene to the appropriate PM. Such an assignment forms a partition over the set of genes and hence we denote this task as the *PM partition identification* (PMPI) problem. PM identification depends on the number of analyzed genes, the number of target PMs, the intrinsic variability of the evolutionary rate for each gene and the intrinsic variability of each PM. The PMPI problem is theoretically and practically hard as it concerns dealing with a lot of data obscured by a massive amount of noise. A possible direction to pursue is to exploit the signal from the data themselves in order to reduce the search space and focus only on relevant partitions. We note that partitioning over the gene set or even different positions in a gene, has been done before [12-14]. However, to the best of our knowledge, these were not based on a rigorous model as the pacemaker rather mainly on the level of the mutation rates.

**Figure 1:**
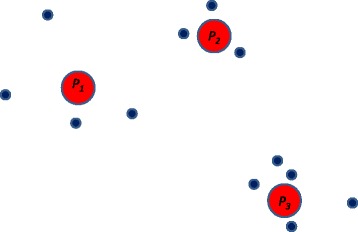
**A graphical illustration of the embedding of the data in Euclidean space.** Red big circles represent pacemakers and the small circles around them - their associated genes.

In this work, a first attempt in this direction is made by devising and employing a novel technique using a series of analytic tools to solve the PMPI problem, and assess the quality of the derived solution. We tackle theoretical computational and statistical issues, as well as challenging engineering obstacles that arise along the way. These include guarantying *homoschedasticity* [15] by working in the log space, removing gene order dependency [16] by employing the Deming regression [17,18], and graph completion through most reliable paths. The result is the partial *gene correlation graph* where edge lengths represent (inversely) correlation, that we subsequently embed into the Euclidean space while preserving the distances (see Figure 1). We apply standard clustering tools to this data and assess the significance of the result. We next formulate the PMPI problem as a recoloring problem [19,20] where a gene’s PM is perceived as its color and the (set of) genes associated with a certain PM form a color class. To measure the quality of partition reconstruction, one may look for the minimum number of genes that need to be recolored in order that every part in the reconstructed partition is monochromatic. This number (the recolored genes) is denoted the *partition distance* [21] and can be solved by a matching algorithm. We however use a greedy maximum weighted matching algorithm, that is practically simpler for implementation and provided very good results empirically. Although theoretically this algorithm provides a 1/2-approximation guarantee for any input, under some statistical conditions the we note, with high probability the correct partition distance is returned.

The simulation results obtained using this approach are highly significant under a random model that we devise. The latter is significant as it implies that we were successful in both extracting the signal from the (noisy) data, and our technique is plausible. Finally, using insights from the simulation analysis, we analyzed the large set of phylogenetic trees of prokaryotic genes that was previously studied in [1]. Because the actual PM partition is unknown, we used the gap statistics criterion of Tibshirani et al. [22] to determine clustering significance and resulting in identification of two distinct genome evolution PMs.

We end this part with a visual illustration given by a graphical representation in Figure 1 where the big red balls represent PMs and the small circles represent genes. By the illustration, it is clear that the more genes and PMs the more likely they are to mix between each other. Similarly, the larger the distance between a gene and its PM, the greater the probability to misclassify it. Conversely with respect to the distance between PMs - the greater this distance, the less likely gene to be misclassified.

## The evolutionary model

Evolutionary history is described by a tree *T*=(*V*,*E*) which is a combinatorial object composed of nodes representing (extant and extinct) species, and edges connecting these nodes such that there are no cycles in *T*. The edges are directed from an *ancestor* to its *descendant* nodes and also correspond to the time period between the respective nodes. There is one node with no ingoing edges, the *root*, and nodes with no outgoing edges are the *leaves* that are labeled by the *species* (or *taxa*) set. Therefore, the topology of *T* indicates the history of speciation events that led to the extant species at the leaves of *T*. Internal nodes correspond to ancestral forms existed at speciation events, and edges indicate ancestral relationships. A node (or a species) is a set of genes *G*={*g*_*i*_} where a gene is a sequence of *nucleotides* of some given length. A gene evolves through a process in which mutations change its nucleotides from one state to another. In our model, all extant and extinct species possess the same set of genes *G*={*g*_*i*_} and all genes *g*_*i*_ evolve along *T* according to an evolutionary model that is assumed to follow a continuous time Markov process. This process is represented by a given rate of mutations *r* per unit of time. In particular, every gene *g*_*i*_ evolves at an intrinsic rate *r*_*i*_∈**r** that is constant along time but deviates randomly along the time periods (i.e. tree edges). Let *r*_*i*,*j*_ be the *actual* (or observed) rate of gene *i* at period *j*. Then $r_{i,j} = r_{i}e^{{\alpha }_{i,j}}$ where $0<e^{{\alpha }_{i,j}}$ is a multiplicative *error factor*. The number of mutations in gene *g*_*i*_ along period *t*_*j*_ is hence *ℓ*_*i*,*j*_=*r*_*i*,*j*_*t*_*j*_, commonly denoted as the *branch length* of gene *g*_*i*_ at period *j*. Throughout, we will use *i* to identify genes *g*_*i*_ and *j* for time periods *t*_*j*_. As the topology of *T* is constant and assumed to be known, we will not make any reference to the tree and regard the edges only as independent time periods *t*_*j*_ for 1≤*j*≤*τ* where *τ*=|*E*|.

We now extend this model to include a pacemaker that accelerates or decelerates a gene *g*_*i*_, relative to its intrinsic rate *r*_*i*_. Formally, a *pacemakerPM*_*k*_ is a set of *τpacesβ*_*k*,*j*_, 1≤*j*≤*τ* where *β*_*k*,*j*_ is the relative pace of *PMk* during time period *t*_*j*_ and −*∞*<*β*<*∞*. Under the UPM model, a gene *g*_*i*_ that is *associated* with PM *P*_*k*_ has actual rate at time *t*_*j*_: $r_{i,j}=r_{i}e^{{\alpha }_{i,j}}e^{{\beta }_{k,j}}$. Hence, for *β*<0 the PM slows down its associated genes, for *β*>0 genes are accelerated by their PM, and for *β*=0, the PM is neutral. Assume every gene is associated with some PM and let *PM*(*g*_*i*_) be the PM of gene *g*_*i*_. Then the latter defines a partition over the set of genes *G*, where genes *g*_*i*_ and $\phantom {\dot {i}\!}g_{i^{\prime }}$ are in the same part if $\phantom {\dot {i}\!}PM(g_{i})=PM(g_{i^{\prime }})$.

### **Comment1**.

It is important to note that gene rates, as well as pace makers paces, are hidden and that we only see for each gene *g*_*i*_, its set of edge lengths *ℓ*_*i*,*j*_.

### **Comment2**.

The presence of two genes in the same part (PM) does not imply anything about their magnitude of rates, rather on their unison of rate divergence.

The above gives rise to the *PM Partition identification Problem:*

### **Problem1**.

*Pacemaker Partition Identification.* Given a set of *n* genes *g*_*i*_, each with *τ* branch lengths {*ℓ*_*i*,*j*_}, the *Pacemaker Partition Identification* (PMPI) problem is to find for each gene *g*_*i*_, its pace maker *PM*(*g*_*i*_).

We first observe the following:

### **Observation1**.

Assume gene *g*_*i*_ has error factor *α*_*i*,*j*_=0 for all time periods *t*_*j*_, 1≤*j*≤*τ* and let *P*^′^=*PM*(*g*_*i*_) be the pace maker of gene *g*_*i*_ with relative paces $e^{{\beta }_{j}}$. Then at all periods *t*_*j*_, $r_{i,j}=r_{i}e^{{\beta }_{j}}$.

Observation 1 implies that if genes *g*_*i*_ and $g_{i^{\prime }}\phantom {\dot {i}\!}$ belong to the same pace maker, and both genes have zero error factor at all periods, then at all periods, the ratio between the edge lengths at each period is constant and equals to $r_{i}/r_{i^{\prime }}$. This however is not necessarily true if one of the error factor is not zero or genes *g*_*i*_ and $g_{i^{\prime }}\phantom {\dot {i}\!}$ do not belong to the same pace maker. Recall that we do not see the gene intrinsic rates (and hence also the ratio between them). However if we see the same ratio between edge lengths across all time periods, we can conclude about the error factors and possibly their belonging to the same PM.

In order to tackle the PM identification problem, we impose some statistical structure (as observed in real data [6]) on the given setting. The goal is to assume that the error factor of each gene is small enough at every period, so that all genes belonging to the same PM, change their actual rate in unison.

Similarly, we assume that *β*_*k*_ varies so that genes from different PMs (parts) can be distinguished (otherwise, no difference except their random error factor exists).

### **Assumption1**.

1. For all genes *g*_*i*_ and periods *t*_*j*_, the gene error factors *α*_*i*,*j*_ follow a normal distribution ${\alpha }_{i,j}\sim N\left (0,{{\sigma }^{2}_{G}}\right)$,
2. For all PMs *P*_*k*_ and periods *t*_*j*_, the PM paces *β*_*k*,*j*_ follow a normal distribution ${\beta }_{k,j}\sim N\left (0,{{\sigma }^{2}_{P}}\right)$,

## The pacemaker partition identification procedure

Here we devise a procedure to solve the PMPI problem that entails a technique to infer distances between genes, constructing the *gene correlation graph*, embed reliably this graph in the plain and apply partitioning algorithms to this embedding. We now describe each of these steps.

### Inferring gene distance

As outlined above, our first task is to infer gene pairwise distances from the raw data, which is gene edge lengths *ℓ*_*i*,*j*_ for every time period (edge) *j*. In particular, as the relevant information is encompassed in the random component of that value, the task of extracting that component is even more challenging.

We now proceed as follows: Given two sets of edge lengths *ℓ*_*i*,*j*_ and ${\ell }_{i^{\prime },j}$ corresponding to genes *g*_*i*_ and $g_{i^{\prime }}$, and time periods *t*_*j*_ for 1≤*j*≤*τ*, we draw *τ* points on a plain $\left ({\ell }_{i,j}, {\ell }_{i^{\prime },j}\right)$. Now, if the error factors, ${\alpha }_{i,j}={\alpha }_{i^{\prime },j}=0$ for all 1≤*j*≤*τ* and we connected all these points, we would obtain a straight line. The slope of that line is the multiplicative factor representing the ratio between the rates of evolution of the corresponding genes - $r_{g_{i}}/r_{g_{i^{\prime }}}$; we denote it ${\rho }_{i,i^{\prime }}$. Obviously, the above description refers to an idealized case. With real data, we never expect to find such a perfect correlation because the characteristic variance ${{\sigma }^{2}_{G}}$ is always non zero. Thus, we expect to find the points scattered around a trend line representing the rate ratio. The density of points around the trend line represents the level of correlation. Our goal is to obtain both the rate ratio ${\rho }_{i,i^{\prime }}\phantom {\dot {i}\!}$ and the level of correlation where the latter will be used to classify between the genes. The method of choice to pursue here is to apply linear regression [15] between the points representing the two edge lengths. There are several outstanding issues that need to be addressed in such a task.

1. **Zero intercept requirement:** Linear regression, when applied to a set of points on a plane, finds a line *y*=*ax*+*b* minimizing the sum of square distances of that line to all the points. As we deal with a multiplicative factor, the trend line has to cross the origin, i.e. *b*=0. Hence we need to modify the standard procedure for regression.
2. **Homoschedasticity requirement:***homoschedasticity* is the property that the error in the dependent variable (*y*) is identically and independently distributed (IID) along the trend line.
  However, by our formulation ${\ell }_{i,j} = t_{j}r_{i,j} = t_{j}r_{i}e^{{\alpha }_{i,j}}$ and the expected value (the value on the trend line) is *t*_*j*_*r*_*i*_. The deviation then is $ t_{j}r_{i}\left (e^{{\alpha }_{i,j}}-1\right)$. As *r*_*i*_ is constant for all time period, we see that the longer the time period *t*_*j*_, the larger the influence of *α*_*i*,*j*_. That is, assume two time periods *j* and *j*^′^ with the same error factor $ {\alpha }_{i,j} = {\alpha }_{i,j^{\prime }}\phantom {\dot {i}\!}$ but different period lengths, WLOG ${\ell }_{j} < {\ell }_{j^{\prime }}$. We obtain different deviations $ t_{j}r_{i}(e^{{\alpha }_{i,j}}-1)< t_{j'}r_{i}\left (e^{{\alpha }_{i,j^{\prime }}}-1\right)$, creating a bias toward longer periods. The following observation follows immediately from the definition of *α*_*i*,*j*_.
  **Observation 2**. *If we take the *$\log {\ell }_{i^{\prime },j} = \log t_{j}r_{i^{\prime }} +{{\alpha }_{i^{\prime },j}}$* we arrive at Homoschedasticity.*
  We denote this as the *log transformation* and also observe the following:
  **Observation 3**. *Under the log transformation the trend line *$\log {\ell }_{i^{\prime },j} = a\log {\ell }_{i,j} + b$* has slope one (a*=1*) and intercept *$b=\log {\rho }_{i,i^{\prime }}$.
  We will use these properties in our calculations.
3. **Gene order independence:** The final problem with the linear regression has to do with the basic assumptions in least squares analysis. In standard least squares, the assumption is that the independent variable *x* is error-free while only the dependent variable *y* deviates from its expected values. In our case, however, the choice between the variables is arbitrary and both are subjected to deviation, according to their characteristic variance ${{\sigma }^{2}_{G}}$. Handling this case with standard least squares would cause arbitrary bias due to the selection of the variables [16]. To handle this case, we apply Deming Regression [17,18]. This approach assumes an explicit probabilistic model for the variables and extracts closed forms expressions (in the observed variables) for the sought expected values. To adjust to our specific case, we will use the observations drawn above. The linear model assumed is of type *η*=*αξ*+*β* where the observations of both *η* and *ξ*, (*x*_1_,…,*x*_*n*_) and (*y*_1_,…,*y*_*n*_), respectively, have normally distributed errors: (i) $x_{i} = \xi _{i}+{\varepsilon }_{x_{i}}\phantom {\dot {i}\!}$, and (ii) $\phantom {\dot {i}\!}y_{i} = \eta _{i}+{\varepsilon }_{y_{i}} = {\alpha }+{\beta }\xi _{i}+{\varepsilon }_{y_{i}}$.
  As can be seen, this is exactly our case. The likelihood function of this model is:
  (1) $$ \begin{aligned} f&={\Huge {\Pi_{1}^{n}}}\left(2\pi{\sigma }^{2}\right)^{-1/2}\exp\left(-\frac {(x_{i}-\xi_{i})^{2}}{2{\sigma }^{2}}\right)\\ &\quad\times\left(2\pi{\sigma}^{2}\right)^{-1/2}\exp\left (-\frac {(y_{i}-{\alpha }-{\beta }\xi_{i})^{2}}{2{\sigma }^{2}}\right) \end{aligned}  $$

Under the general formulation, the ML value for *α* is: ${\alpha }= \bar x +\bar y{\beta }$ where $\bar x$ and $\bar y$ are the average values for *x*_*i*_ and *y*_*i*_. However, in our formulation we have *β*=1 and hence ${\alpha } =\bar x + \bar y$. Having *α* at hand, we can reconstruct the trend line and obtain the deviation of every point from it. Finally, by our formulation, ${\rho }_{i,i^{\prime }}\phantom {\dot {i}\!}$ is given by *exp*(*α*) and the correlation between the rates is the standard *sample Pearson correlation coefficientr*(*X*,*Y*) [15]:

(2) $$ r = \frac{\sum_{i=1} \left(X_{i}-\bar X\right)\left(Y_{i}-\bar Y\right)}{\sqrt{\sum_{i=1}\left(X_{i}-\bar X\right)^{2}}\sqrt{\sum_{i=1}\left(Y_{i}-\bar Y\right)^{2}}}.  $$

### The gene correlation graph

After we inferred all pair-wise correlations, we can build the *Gene Correlation GraphG*=(*V*,*E*,*w*) aiming at representing the correlation between the pairs of genes. *V*={*g*_*i*_} and an edge (*i*,*i*^′^)∈*E* if *r*(*i*,*i*^′^) from Equation (2) is greater than some threshold *δ*_*r*_, maintaining a minimal level of correlation in the graph. Hence we set *w*(*i*,*i*^′^)=*r*(*i*,*i*^′^) and as −1≤*r*≤1 we are guaranteed no negative weighted edges exist. Note that we are not interested in *r*^2^ which may reflect high *negative* correlation, rather only in high positive correlation.

Recall that our initial goal was to partition genes into clusters (PMs) according to correlation. Perhaps the most commonly used technique is *k-means* [23,24] that aims at minimizing the within-cluster sum of squares (WCSS). These techniques operate in the Euclidean space and hence some distance preserving technique is required to embed the correlation graph *G* in the space. Multidimensional Scaling [25] (also Euclidean embedding) is a family of approaches for this task. Kruskal’s iterative algorithm [26] for non-metric multidimensional scaling (MDS) receives as input a (possibly partial) set of distances and the desired embedding should preserve the *order* of the original distances. It requires however a full matrix as a starting guess.

Our approach here is to join every two nodes by the most reliable connection and with the highest correlation. This translates to finding the path with the minimum number of nodes (hops) and that the multiplication of the corresponding weights is minimal. This distance measure, min hop min weight (MHMW), is also useful in communication networks, where hop distance corresponds to reliability [27].

While the naive algorithm for the latter runs in time *O*(*n*^3^) it can be easily seen that we can solve the problem in time *O*(*n*^2^ log*diam*(*G*)) where *diam*(*G*) is the diameter of *G*. The completed graph $\hat G$ serves as input to the *Classical multidimensional scaling* (CMDS) [28] whose output serves as the initial guess to the Kruskal’s non-metric MDS. Once we have the embedding, we can apply k-means and obtain the desired clustering.

Below is the complete formal procedure *PMPI*:

**Procedure*PMPI(, δ***_***r***_***)*:**

1. Set the correlation graph *G*=(*V*,*E*) with *V*=*∅*, *E*=*∅*
2. *V*={*g*|*gisagenein*}
3. for all *g*_*i*_,*g*_*j*_∈
  apply the Deming regression between *g*_*i*_ and *g*_*j*_ to determine *r*(*g*_*i*_,*g*_*j*_)
  if *r*(*g*_*i*_,*g*_*j*_)≥*δ*_*r*_, then add {(*g*_*i*_,*g*_*j*_)} to *E* and set *w*(*g*_*i*_,*g*_*j*_)←*r*(*g*_*i*_,*g*_*j*_)
4. $\hat G \leftarrow MHMW(G)$
5. apply Classical Multidimensional Scaling (cmdscale) to the full graph $\hat G $
6. apply Kruskal’s iterative algorithm (isoMDS) to the original distance matrix, starting from cmdscale output
7. apply *kmeans* to the resulted embedding

## Simulation analysis

In order to evaluate the PMPI procedure described in Section 1 and derive practical intuition over our model, we performed simulation according to the basic lines described above.

In a simulation study, a crucial part involves the assessment of the reconstruction quality with respect to the model on which the input was generated. As the PMPI is targeted at reconstruction of the original PM partition, we chose to use the partition distance measure.

### Partition distance

Once we obtain the reconstructed clustering, it should be compared to the original, model clustering. The task of comparing two clusterings can be casted as a partition distance where every clustering is a partition over the element set. We now define it formally. For two sets *s*_*i*_ and *s*_*j*_, the distance *d*(*s*_*i*_,*s*_*j*_) is the size of their symmetric difference set *s*_*i*_△*s*_*j*_=(*s*_*i*_∖*s*_*j*_)∪(*s*_*j*_∖*s*_*i*_). Analogously, the *similaritys*(*s*_*i*_,*s*_*j*_) is the size of their intersection set *s*_*i*_∩*s*_*j*_ and it is easy to see that given the sizes of the two sets, one is derived from the other. A *partition* over a ground element set *N* is a set of parts {*p*_*i*_} where every part is a subset of *N*, {*p*_*i*_} are pairwise disjoint (i.e. *p*_*i*_∩*p*_*j*_=*∅* for every *i*≠*j*), and their union is *N*. The cardinality of , denoted as $|\mathcal {P}|$ is the number of parts. A partition can also be perceived as a *coloring* function *C* from *N* to a set of colors  (the color classes) where *C*(*x*) is the part of element *x*∈*N* under partition *P* (or equivalently *C*). Henceforth we will use the notions of PM identity and a color interchangeably. Given two partitions  and $\mathcal {P}'$ over the same element set *N* (or equivalently *C* and *C*^′^), denoted as the *source* and *target* partitions, we are interested in their *partition distance*$d\left (\mathcal {P},\mathcal {P}'\right)$ as some measure of similarity. The simplest approach is naturally the number of elements with different colors at the two partitions, i.e., *x*∈*N* s.t. *C*(*x*)≠*C*^′^(*x*), and we call it the *identity similarity*. Under this approach, the partition distance between  and $\mathcal {P}'$, $d\left (\mathcal {P},\mathcal {P}'\right)$, is defined as:

(3) $$ d\left(\mathcal{P},\mathcal{P}'\right) = \sum_{x\in N} \bar {\delta }(C(x),C'(x))  $$

where $\hat {\delta }$ is the *inverse Kronecker delta*:

(4) $$ \bar {\delta }(i,j) = \left\{ \begin{array}{ll} 1 & \text{if}\; i \ne j\\ 0 & \text{otherwise.} \end{array} \right.  $$

This of course is simple and is an upper bound on a more accurate approach: colors can be permuted between the two partitions, in the sense that a color is mapped by a function *f* to another color in  and now $d\left (\mathcal {P},\mathcal {P}'\right)$ is defined as

(5) $$ d\left(\mathcal{P},\mathcal{P}'\right) = \sum_{x\in N} \bar {\delta }\left(\,f(C(x)),C'(x)\right).  $$

It is easy to see that under this definition, *f* in the first approach is simply the identity function *f*(*c*)=*c* for every $c\in \mathcal {C}$. This essentially defines a *recoloring problem* [20] where the goal is to recolor the least number of elements in $\mathcal {P}'$ (or *C*^′^) such that *f*(*C*(*x*))=*C*^′^(*x*) for every element. Hence the cost of *f* is the number of elements *x* s.t. *f*(*C*(*x*))≠*C*^′^(*x*).

**Figure 2:**
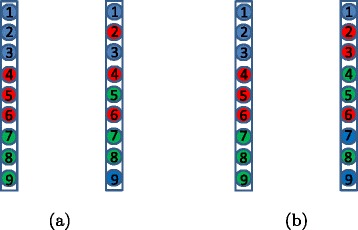
**Partition similarity under two mappings.** **(a)** The identity similarity is the optimal where we just count the number of recolored vertices. **(b)** The optimal similarity is obtained by mapping the blue color to red, the red to green, and green to blue, yielding partition distance 3.

Figure 2 illustrates the idea. In the left side, most of the genes chose their original PM (color) and therefore the identity similarity is the optimal mapping. However, in the right side, most of the blue genes chose the green PM, most of the red genes chose the blue PM, and most of the green genes chose the red PM. Therefore this is also the optimal mapping.

Now, since the mapping is from  to , *f* is a bijection or simply a *matching* between the set of colors. In [21] Gusfield noted that the partition distance problem can be casted as an assignment problem [29] and hence be solved by a maximum flow in a bipartite graph in time *O*(*mn*+*n*^2^ log*n*) [30]. Matching problems are among the most classical and well investigated in theoretical, as well as in practical, computer science [31]. Although it has a polynomial time exact algorithms with many flavors [30], a host of works on approximated solutions were introduced. For its very simple implementation and empirically accurate results that are based on theoretical properties we show below, we chose to use a very simple greedy algorithm, named *Greedy PartDist*. The algorithm works recursively and, at each recursion, chooses the heaviest edge (*u*,*v*) in the graph, adds it to the matching *M* and removes from the graph all other edges (*u*,*v*^′^) and (*u*^′^,*v*) for *u*^′^,*v*^′^∈*V*. The algorithm is given below.

***Greedy PartDist***$\left (\mathcal {P},\mathcal {P}'\right)$***:***

- Construct the bipartite graph $B\left (\mathcal {P},\mathcal {P}',E,w\right)$ with *w*(*p*,*p*^′^)=*s*(*p*,*p*^′^) for every $p\in \mathcal {P}$ and $p'\in \mathcal {P}'$ s.t. *s*(*p*,*p*^′^)>0
- sort *E* according to *w* by descending order and use it as a stack to fetch elements.
- *M*_*G*_←*∅*
- while *E* is not empty
  - (*p*_0_,*p*0′)←*pop*(*E*)
  - *M*_*G*_←*M*_*G*_∪(*p*_0_,*p*0′)
  - Remove all (*p*_0_,*p*_*j*_) and (*p*_*i*_,*p*0′) items from *E*
- match arbitrarily zero-degree elements from  with zero-degree elements from $\mathcal {P}'$ and add to *M*_*G*_
- Return *M*_*G*_

#### ***Claim***.

The algorithm *Greedy PartDist* terminates in time *O*(*m* log*n*).

#### *Proof*.

The algorithm is comprised from the following major tasks:

- **building the graph*B*** - Every part $p'\in \mathcal {P}'$ holds the number of elements it has from every $p\in \mathcal {P}$, hence in *O*(*E*)*B* is constructed.
- **Sorting*E*** - *O*(*m* log*m*)=*O*(*m* log*n*^2^)=*O*(*m* log*n*) by any standard sorting procedure.
- **Constructing*M***_***G***_ - if we go downwardon *E* and additionally holding an auxiliary link from a part to all its edges, each element in *E* is accessed only once and in constant time, *O*(*m*) in total.
  The final stage consists of matching orphan parts from both partitions. It can be easily perceived that this can be done in time linear in *n*.

This algorithm provides a 1/2-approximation guarantee [32]. In the Appendix we provide the same approximation guarantee (i.e. 1/2) by the generic recursive analysis of the *local ratio* technique [33].

#### The greedy algorithm under the stochastic models

It is interesting to analyze the performance of the greedy algorithm under our stochastic model. It is easy to see (even simply for symmetry arguments) that under our model assumption, every gene remains in its part with probability *α* (that depends on the two variances *σ*_*P*_ and *σ*_*G*_) and with probability 1−*α* chooses uniformly a partition (including its own partition). The expected identity similarity here is the sum over the elements maintaining their part plus those randomly chose that same (original) part.

We therefore obtain:

(6) $$ E[s_{{{{\alpha }-u}}}\left(\mathcal{P},\mathcal{P}'\right)] = \frac {(k-1){\alpha } n}k+\frac nk  $$

##### **Definition1**.

We say that a PM *P* is *correctly clustered* if most of the genes associated with *P* as a source PM, choose *P* as their target PM.

Definition 1 is demonstrated visually in Figure 2(a) where every color is preserved (in the right side) by most of its vertices. Definition 1 implies that under a correctly clustered PM, a significant core set of genes stay together is the target PM (part). It is easy to see that, under our stochastic model, if enough genes are associated with every source PM, then all PMs are correctly clustered.

##### ***Claim***.

Assume every color is correctly clustered. Then Algorithm Greedy PartDist returns the correct result.

##### *Proof*.

The proof follows by induction on the number of PMs ||. For a single PM, there is a single edge in the bipartite graph and this edge is chosen. For $|\mathcal {P}|>1$, note that by the assumption, the heaviest edge emanating from each PM (node) in to its corresponding color in the partition $\mathcal {P}'$. In particular, this is true for the heaviest edge in the bipartite graph, linking between the nodes corresponding to some PM *P*. Then the algorithm chooses that edge and remove all edges adjacent to it. Therefore, PM *P* was correctly chosen and by the induction hypothesis the algorithm returns the correct result.

### Simulation results

To asses the effectiveness of our PM partitioning identification procedure *PMPI*, we conducted the following simulation study. Number of genes *n* was held constant *n*=100 giving rise to ${100 \choose 2}=4950$ pairs of correlation tests. The number of edges per a gene tree was set to 25, reflecting the average size of the agreement tree among our real data trees. To simulate low agreement similarly to our real data (low MAST value) we discarded every pair with probability 2/3 maintaining approximately 1/3 of the pairs (see more details in Section ‘Results on real data’). Every PM *P*_*k*_ was associated with an intrinsic variance ${{\sigma }^{2}_{P}}$ that sets its relative pace to $e^{{\beta }_{k,j}}$ where ${{\beta }_{k,j}} \sim N\left (0,{{\sigma }^{2}_{P}}\right)$. Similarly, every gene sets its rate at period *j* to $r_{i,j}=r_{i}e^{{\alpha }_{i,j}}e^{{\beta }_{k,j}}$ where ${{\alpha }_{i,j}} \sim N\left (0,{{\sigma }^{2}_{G}}\right)$ (See Model Section 1 for full details).

Every gene was associated with a source PM, same number of genes for each PM. Number of PMs *k* varied from 2 to 10 (i.e. 10 to 50 genes per PM). Distance between genes was set as 1−*r* from the regression line where the latter was derived by the Deming regression.

This has defined our correlation graph described above.

In order to apply clustering algorithms on the elements, the elements need to be embedded in some Euclidean space. Multidimensional scaling takes a set of dissimilarities (over a set of elements) and returns a set of points in a Euclidean space, such that the distances between the points are approximately equal to the dissimilarities. A set of Euclidean distances on *n* points can be represented exactly in at most *n*−1 dimensions. The procedure *cmdscale* follows the analysis of Mardia [34], and returns the best-fitting *k*-dimensional representation, where *k* may be less than the argument *k* (and by definition smaller than *n*). In our implementation, in order to avoid any distortion, we set *k* to the maximum value as determined by the data (and is found and returned by the method). We used a version of *cmdscale* that is implemented in R. As *cmdscale* requires a complete graph, we used the min-hop-min-weight (MHMW) algorithm. The output of the MHMW is a complete graph where the weight between any two points is the lightest (min weight) path among all min hop reliable paths (paths between trees for which correlation was derived). At this point we can use *cmdscale* to map this graph to the Euclidean space. Note however, that this mapping corresponds *not* to the original graph, rather to some approximation of it derived by the output of the MHMW algorithm. This mapping however serves as an initial guess to the iterative mapping of the original, partial, distance matrix. This iterative process is done by the function *isoMDS* implemented in R. This mapping will serve us for the clustering operation. Now, as opposed to real data, here we know the original number of clusters, we can just set this as the number of clusters required. We used *kmeans* implemented by R to obtain the optimal clustering.

**Figure 3:**
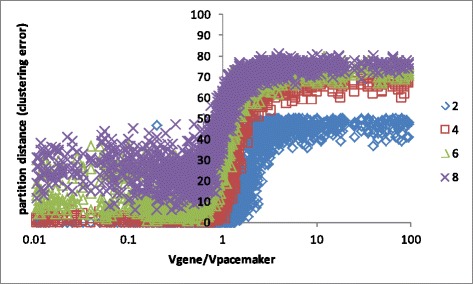
**Partition distance obtained by applying the PMPI technique on simulated data versus the gene/pacemaker variance ratio; the plots are shown for 2, 4, 6 and 8 clusters (PMs).**

Our results appear in Figure 3. The measured quantity is (normalized) partition distance as measured by our *greedy PartDist*. The independent variable is the ratio between *σ*_*G*_ and *σ*_*P*_. The larger *σ*_*P*_ the more dispersed are the PMs and hence farther from one another. Equivalently, the smaller *σ*_*G*_, the more concentrated around their PM are the genes. Therefore, we expect that the smaller the ratio *σ*_*G*_/*σ*_*P*_ is, i.e. PMs are spaced away from each other while their associated genes are more concentrated, we get better results in the sense that more genes remain in their original cluster and successfully identified. Also, we expect that the larger the number of PMs, the greater the mixing between them with genes end up in PMs that are neighboring to their original PMs. Indeed it can be seen that for two and four PMs, for any ratio of *σ*_*G*_/*σ*_*P*_≤1 a very accurate reconstruction is achieved and so as to six clusters, but for ratio a little less than 1. It is also shown that for every number of PMs, at some critical *σ*_*G*_/*σ*_*P*_ ratio (that depends on #PMs) the reconstruction curve reaches a saturation that tends to the random similarity as we computed above.

## Results on real data

**Figure 4:**
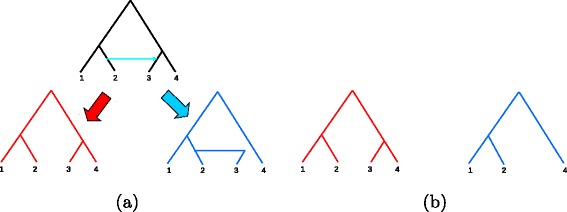
**Cases in which agreement subtrees must be taken.** **(a)** HGT case: An HGT event occurred in the blue gene, from the edge leading to 2 to the one leading to 3. While the red gene is unaffected, the blue gene has 2 and 3 as sister taxa. **(b)** Missing taxa case: Taxon 3 is missing the blue gene. In both cases (a) and (b), a tree over taxa {1,2,4} has the same topology.

Working with real data poses some other serious problems requiring solution. The first, is that we don’t have here exactly *τ* periods with edge length *ℓ*_*i*,*j*_ for every gene *g*_*i*_ rather a set of trees with loose pairwise agreement. This loose agreement is due to vast discordance between the histories of the various genes as a result of phenomena such as horizontal gene transfer (HGT) or incomplete lineage sorting (ILS, see more details below). However, discordance can arise even from the simple fact that some gene is missing in some specific species, resulting in a contraction of internal nodes. Figure 4 illustrates HGT and missing taxa cases. In both cases, the taxa set {1,2,4} is an agreement subset for both gene-trees and in this case also maximal agreement subset.

To cope with this problem, we employ the idea of Maximum Agreement Subtrees (MAST) [35], that seeks for the largest subset of species under which the two trees are the same. Under MAST (or in general, any subset of the leaf set), edges not connecting any species to the induced tree, are removed, and internal nodes with degree two are contracted, while maintaining the original length of the path. Hence for every pair of genes (trees) we need to find the MAST and compare lengths of corresponding edges.

Additionally, here as opposed to a simulation study, we do not know the “real” partition and cannot compare the resultant clustering to it. Therefore, another method for assessing the results should be employed. Here we need to compare the result to the probability of being obtained under a random model. Recall that at the final stage of the *PMPI* procedure we employ the *kmeans* algorithm which seeks to minimize some *error measureW*_*K*_. This error measure holds the sum of all pairwise distances between members of the same cluster, across all clusters in the partition. It is clear that the more clusters, the smaller *W*_*K*_ is. However, the decrease in *W*_*K*_ is the largest near the real value of the number of clusters *k*=*K*, and vanishes slowly for *k*>*K*. Therefore, a threshold for the improvement (decrease) in *W*_*K*_ must be defined as a stopping condition, above which we don’t increase the number of clusters *k*. The *gap statistics analysis* [22] compares the improvement in *W*_*K*_ under the real data, to that of a random model. The gap (between the improvements) forms an “elbow” at the optimal (real) *K* and this is the stopping condition.

**Figure 5:**
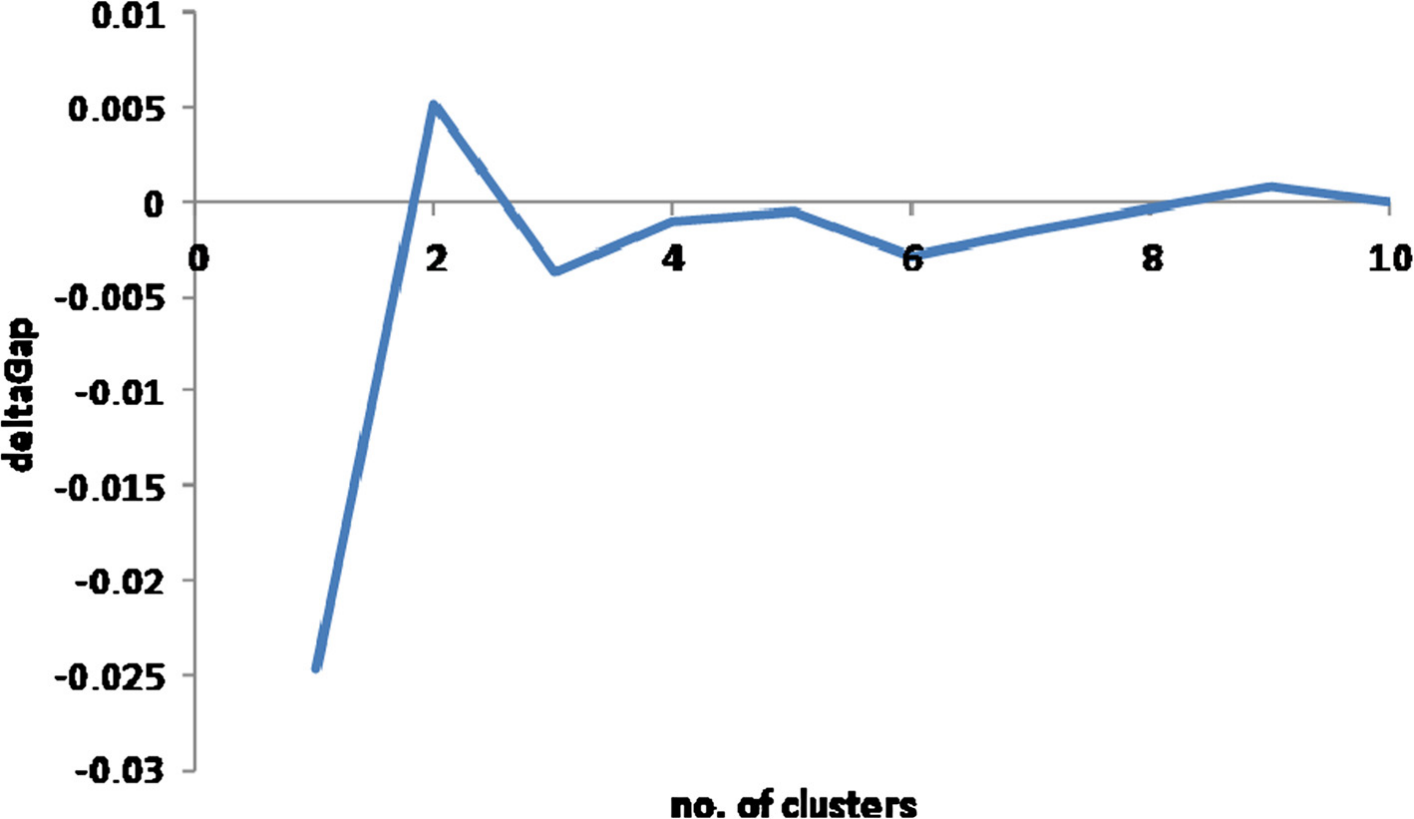
**The deltaGap function for 2755 analyzed genes, k from 1 to 10.** According to Tibshirani et al. [22], the smallest k producing a non-negative value of deltaGap[k] = Gap[k]-Gap[k+1]+sigma[k+1] indicates the optimal number of clusters.

The real data we chose to analyze is the one used by us [1] previously, of a set of gene trees that covers 2755 orthologous families from 100 prokaryotic genomes [36]. Prokaryotic evolution is characterized by the pervasive phenomena of horizontal gene transfer (HGT) [37,38], resulting in different topologies for almost any two gene trees. To account for this we employed the MAST procedure for every gene pair and considered this pair only if the MAST contained at least 10 leaves (species). Branch lengths of the original trees were used to compute the branch lengths of the corresponding MAST components (by computing path lengths). The variant of Deming regression in the log space as described in Section ‘Simulation analysis’ was performed on the logarithms of the lengths of equivalent branches in both MAST components. The standard sample Pearson correlation coefficient was used as the measure of correlation between the branch lengths. The graph of correlations between the gene trees contained a giant connected component containing 2755 genes and 1,250,972 edges, 33% of the maximum possible number (an edge in the graph exists only when the MAST for the corresponding pair of trees consists of at least 10 species). To cluster these genes according to the correlation between their branch lengths, the data were projected using isoMDS into a 30-dimensional space based on the sparse matrix where 1−*r* (correlation coefficient) was used as a distance. We ran *k-means* for *k* spanning the range from 2 to 30. The random model we chose to consider is the fully random uniform model (i.e., *α*=0, no advantage to source PM) and we compared the results to this model. Grouping these 2755 genes in two clusters containing 1550 and 1205 members, respectively, yields the optimal partitioning according to the gap function statistics (Figure 5). We see the typical “elbow” at the value of *k*=2. The absolute results were 5,587,960 for the total graph weight, 2,686,914 and 2,285,921 weight within each of the clusters, and 615,125 between them. Analysis of the cluster membership reveals small albeit significant differences in the representation of functional categories of genes but no outstanding biologically relevant trends were detected. Therefore, we can hypothesize that if indeed the data gives rise to multiple PMs, this signal is completely obscured by the amount of noise produced by the genes themselves (i.e. loose adherence to the associated PM), and noise introduced by artificial factors such as MAST, multiple sequence alignment, and phylogenetic reconstruction.

## Conclusions

The universal pacemaker (UPM) model provides a more general framework to analyze genome evolution than the MC model as it makes no assumptions of the absolute evolutionary rates of gene, only on the relative rates. This provides a better explanation to the data observed at extant species. However, similarly to the MC, the UPM is extremely over-dispersed, with the noise complicating detailed analysis. The difficulty in PM analysis is caused both by the weak informative signal and by the large volume of the data.

A natural expectation, however, is for different gene groups, to adhere to different PMs, characterized by different functions. This classification imposes a partition over the gene set where each gene is associated with its own PM. The inference of such a partition is challenging twofold; first from information perspective, as it needs to overcome a high level of “noise”, both biological, as well as artificial. Next, the computational task of solving the PMPI problem requires investigating all possible partitions over the gene set.

In this work we provide the first heuristic procedure for detecting such a partitioning that is based on theoretical ground. We use the Deming regression to infer correlation between pairs of genes, and represent this correlation relationship in a graph. Subsequently, we embed this graph in the Euclidean space and apply a clustering procedure to it.

We also provide simulation and empirical results of the application of this procedure. In the simulation study, we have shown that the proposed procedure is sound and is capable of detecting the original partition with high accuracy for a fairly small (up to 6) number of PMs as long as the intrinsic gene rate variance is at the size of the PM variance. In the real data realm, we succeed in showing that the analyzed genome-wide set of gene trees is optimally partitioned between two PMs, and the improvement in the statistical explanation is small albeit highly significant. The partition of different functional gene groups between the two PMs is also statistically significant (WRT random partitioning of each group) however the biological interpretation of this partitioning is challenging and remained for future research.

## Appendix

### Performance guarantees for greedy PartDist algorithm

We now prove a performance guarantee on the output matching returned by the *Greedy PartDist* algorithm. As noted above, it was already shown [32] that the algorithm provides a 1/2-approximation, however the approach used here is different and of separate interest. The first result applies to a general input and hence should be compared to the optimal solution for such cases that is obtained by solving the assignment problem.

#### A 1/2-approximation maximum weight perfect matching via local ratio

Our approximation algorithm makes use of the local ratio technique, which is useful for approximating optimization covering problems such as vertex cover, dominating set, minimum spanning tree, feedback vertex set and more [33,40,41]. We hereafter describe it briefly, in the context of our setting: The input to the problem is a triplet $\left (B=\left (\mathcal {P},\mathcal {P}',E\right),f: 2^{E}\rightarrow \{0,1\}, w:E\rightarrow \mathbb {R}^{+}\right)$ where $B=\left (\mathcal {P}, \mathcal {P}', E\right)$ is a bipartite graph, *f* maps (edge subsets) to the set of valid solutions and let *Σ*={*E*^′^⊆*E*:*f*(*E*^′^)=1} be that set. The goal is to find a subset *X*∈*Σ* such that *w*(*X*) is maximized, i.e. $w(X)=OPT(V,\Sigma,w)=\max \limits _{Y\in \Sigma }w(Y)$ (in our context *E* is the set of edges, and *Σ* is the set of perfect matchings in *B*). The local ratio principle is based on the following observation (see e.g. [33]):

##### **Observation4**.

For a maximization problem *π* and every two weight functions *w*_1_,*w*_2_:

$${OPT}_{\pi}(E,\Sigma,w_{1})+{OPT}_{\pi}(E,\Sigma,w_{2}) \geq {OPT}_{\pi}(E,\Sigma,w_{1}+w_{2}) $$

Now, given our initial weight function *w*, we select *w*_1_,*w*_2_ s.t. *w*_1_+*w*_2_=*w* and |*support*(*w*_1_) |<|*support*(*w*)| where *support*(*w*)={*i* : *w*(*i*)>0} (i.e. *w*_1_ is relevant to a smaller set). We first apply the algorithm to find an *r*-approximation to (*E*,*Σ*,*w*_1_) (in particular, if *support*(*w*_1_)=*∅*, then every matching is an optimal matching to (*E*,*Σ*,*w*_1_)). Let *X*_1_ be the solution returned for (*E*,*Σ*,*w*_1_), and assume that *w*(*X*_1_)≥*r*·*OPT*(*E*,*Σ*,*w*_1_). If we could also guarantee that *w*(*X*_2_)≥*r*·*OPT*(*E*,*Σ*,*w*_2_) then by Observation 4 we are guaranteed that *X*=*X*_1_∪*X*_2_ is also an *r*-approximation for (*E*,*Σ*,*w*_1_+*w*_2_=*w*). In order to guarantee this we first need to care that the solutions *X*_1_ and *X*_2_ comply. That is, the joint solution composed by *X*_1_ and *X*_2_ is a valid solution (in the simple, unweighted case, this is just a union of *X*_1_ and *X*_2_). We therefore devise the following decomposition of *w* to *w*_1_ and *w*_2_:

##### **Definition2**.

For a weighted bipartite graph $B=\left (\mathcal {P},\mathcal {P}',E,w\right)$ let *e*_*M*_=(*p*_*M*_,*pM*′)∈*E* be the maximal weight edge. Then set

(7) $$ {\fontsize{8.1}{12}{\begin{aligned} w_{2}(e) = \left\{\!\!\begin{array}{ll} w(e)\! & \!\text{if} \;e\,=\,\left(p_{M},p_{j}\right) \text{or}\; e\,=\,\left(p_{i},p'_{M}\right) \text{for every}\; p_{i} \!\in\!\mathcal{P} \text{and}\, p_{j}\!\in\!\mathcal{P}' \\ 0 & \text{otherwise.} \end{array} \right. \end{aligned}}}  $$

That is, *w*_2_ leaves the weight of all edges adjacent to either *p*_*M*_ or *pM*′ intact, and sets to zero all other edges in *support*(*w*). We also let *w*_1_(*e*)←*w*(*e*)−*w*_2_(*e*).

We now analyze the algorithm *Greedy PartDist* above. We prove this by two observations.

##### **Observation5**.

Let *w*_2_ be as defined above. Then, at every recursion, the algorithm *Greedy PartDist* provides a $\frac 12$-approximation to *support*(*w*_2_).

##### *Proof*.

Since at most two edges from *support*(*w*_2_) are present in an optimal matching *M*^∗^, by taking the heaviest edge *e*_*M*_=(*p*_*M*_,*pM*′) the observation follows.

##### **Observation6**.

Let *X* be the matching returned by the algorithm upon return from the recursion, i.e. *X* is a matching for $B=(\mathcal {P},\mathcal {P}',E,w_{1})$ and assume *X* is at least a $\frac 12$ approximation for *support*(*w*_1_). Then *X*∪*e*_*M*_=(*p*_*M*_,*pM*′) is a valid matching with weight at least *w*(*M*^∗^)/2.

##### *Proof*.

The first part of the claim follows as the set of edges are disjoint and cover all nodes in *support*(*w*). The guarantee on the approximation follows by a simple induction on the number of iterations. The basis is when *support*(*w*)=*∅*. The induction step follows easily using Observation 5.
